# Relationship between cognitive dysfunction and urinary 8-OHdG levels in children with obstructive sleep apnea

**DOI:** 10.3389/fneur.2025.1502906

**Published:** 2025-03-25

**Authors:** Bolin Chen, Jiapeng Ji, Meng Lv, Xueyun Xu, Yuqing Wang

**Affiliations:** Department of Respiratory, Children’s Hospital of Soochow University, Suzhou, China

**Keywords:** obstructive sleep apnea, cognitive dysfunction, children, 8-OHdG, biomarker

## Abstract

**Background:**

Obstructive sleep apnea (OSA) is a condition characterized by partial or complete obstruction of the upper respiratory tract during sleep, which can result in neurocognitive deficits and cognitive dysfunction in children. Oxidative stress may play a significant role in OSA-related disorders, with 8-hydroxy-2′-deoxyguanosine (8-OHdG) serving as a primary marker of oxidative DNA damage for assessing oxidative stress levels. This study aims to investigate the relationship between urinary 8-OHdG levels in children with OSA and cognitive dysfunction.

**Methods:**

The study included children with habitual snoring from April 2023 to June 2024 at the Children’s Hospital of Soochow University. All participants completed the PedsQL questionnaire and underwent polysomnography (PSG) assessment and urine collection for 8-OHdG analysis.

**Results:**

In total, 99 children with OSA and 35 children with non-OSA were included. The urinary 8-OHdG levels were higher in the OSA group (240.94 ± 11.51 pg./mL) than in the non-OSA group (230.73 ± 13.82 pg./mL) (*p* < 0.001). Moreover, 8-OHdG was correlated with the course of the disease, obstructive apnea–hypopnea index, minimum arterial oxygen saturation (SaO2), average SaO2, oxygen desaturation index, and cognitive dysfunctions evaluated by the PedsQL questionnaire (all *p* < 0.05). The area under the receiver operating characteristics curve of 8-OHdG was 0.661 (95%confidence interval, 0.550–0.773). Binary logistic regression analysis revealed that 8-OHdG was significantly associated with the School Functioning score (*p* = 0.004).

**Conclusion:**

Urinary 8-OHdG may serve as a non-invasive, objective biomarker for assessing cognitive dysfunctions in children with OSA.

## 1 Introduction

Obstructive sleep apnea (OSA) is distinguished by persistent partial upper airway obstruction or intermittent complete obstruction (obstructive apnea) that disrupts normal ventilation during sleep and normal sleep patterns (1). Studies indicate that long-term, repeated hypoxemia in children with OSA can lead to neuropsychological deficits affecting neurocognitive functions such as attention, memory, executive function, and alertness (2). Cognitive impairment in children has caused widespread concern and anxiety among parents (3) and has begun receiving increasing attention. Furthermore, the assessment of cognitive function in children often relies on diverse scales, including the Wechsler Abbreviated Scale of Intelligence (4), Wechsler Intelligence Scale for Children, Fourth Edition (WISC-IV) (5), the Children’s Memory Scale (5), California Verbal Learning Test (5), the Kaufman Assessment Battery for Children (6), and the Pediatric Quality of Life Inventory (PedsQL) questionnaire (7). A wide variety of neuropsychological measures potentially introducing subjectivity into the findings due to individual differences in children’s compliance with examiners when employing these scales. Consequently, the development of objective and easily measurable biomarkers holds significant value for practical clinical applications.

The characteristic of OSA is recurrent episodes of hypoxia–reoxygenation. Oxidative stress plays a significant role in the etiology of sleep apnea–related disorders (8). Reactive oxygen species (ROS) are byproducts of oxygen’s natural metabolic conversion within cellular processes. In high-stress scenarios, a significant surge in ROS can surpass antioxidant defenses, resulting in oxidative stress (OS). This condition impairs cellular integrity through molecular damage, potentially triggering DNA alterations, apoptotic cell death, and inflammatory responses (9). The brain’s vulnerability to oxidative stress has been recognized as a pivotal factor contributing to cognitive decline owing to its distinct characteristics such as high oxygen utilization, abundant presence of polyunsaturated fatty acids, and a relative deficiency in antioxidant defenses compared to other body tissues (10). 8-Hydroxy-2′-deoxyguanosine (8-OHdG), one of the major forms of oxidative deoxyribonucleic acid (DNA) damage, is used to evaluate the oxidative stress level. Moreover, the increase in 8-OHdG is significantly correlated with acute ischemic stroke and Alzheimer’s disease (11, 12). A wealth of data from animal and *in vitro* studies indicates that the buildup of reactive oxygen species can initiate numerous biological processes, including amyloid beta deposition and DNA damage, which are pivotal in the development of cognitive decline (13). In children with obstructive sleep apnea, those exhibiting cognitive impairment have higher levels of 8-OHDG in their morning urine compared to those without cognitive dysfunction (14). Nonetheless, the majority of investigations into the association between OSA and 8-OHDG have predominantly centered on adult populations. For instance, Peres BU (15) conducted polysomnography and measured plasma 8-OHdG levels in 402 patients with an average age of 50. The relationship between OSA and 8-OHdG was evaluated both before and after controlling for confounding factors. Similarly, Yamauchi M (16) performed polysomnography and measured urinary 8-OHdG levels in 102 patients with an average age of around 50, demonstrating a correlation between the severity of OSA and oxidative stress. The domain concerning children remains significantly understudied, and the correlation between urinary 8-OHDG levels and the extent of cognitive dysfunction in children has not been exhaustively examined. Therefore, the aim of this study is to compare the levels of 8-OHdG in children with obstructive sleep apnea with or without cognitive dysfunction and explore the correlation between these levels and clinical and cognitive parameters.

## 2 Materials and methods

### 2.1 Study design and participants

Between April 2023 and June 2024, a total of 131 consecutive participants (99 children with OSA and 35 children with primary snoring [PS]) who were admitted to the Children’s Hospital of Soochow University because of habitual snoring during sleep were enrolled. All participants underwent polysomnography and completed the Pediatric Quality of Life Inventory (PedsQL) questionnaire.

This study was approved by the ethics committee of the Children’s Hospital of Soochow University (approval no. 2023CS058). All children who participated in the research were accompanied by parents who provided written informed consent. The inspection items and processes involved in this study are in line with the Declaration of Helsinki.

### 2.2 Inclusion and exclusion criteria

In this study, inclusion was considered according to the following criteria: (1) Children aged 3 to 14 who have undergone polysomnography (PSG) and urine examinations and whose parents have completed the Pediatric Quality of Life Inventory (PedsQL) assessment tool; (2) Children who exhibited clinical manifestations such as snoring, mouth breathing, and suffocation during nighttime sleep, empty their bladder and stool before falling asleep, and have no habit of urinating or defecating at night; (3) On the day of polysomnography examination, children did not engage in vigorous exercise, completed meals 2 h before the examination, and had no intake of food, beverages, sedatives, or sleeping pills except for drinking water before falling asleep. The exclusion criteria were as follows: Children (1) who have undergone adenoidectomy and/or tonsillectomy, oral orthodontic treatment, etc.; (2) with combined facial deformities, such as the small jaw, short jaw, and macroglossia; (3) with acute or chronic respiratory infections, cardiovascular and pulmonary diseases, hematological diseases, neuromuscular diseases, or other diseases that can cause nocturnal hypoxemia; (4) who use psychotropic or sedative medicine that may affect memory or sleep; (5) with neurological abnormalities based on medical history, radiological examination, or electroencephalogram; and (6) with other sleep problems such as insomnia, parasomnia, narcolepsy, restless legs syndrome, or abnormal movement during sleep.

### 2.3 Sleep studies

The PSG outcomes were meticulously documented, encompassing key metrics such as the Obstructive Apnea-Hypopnea Index (OAHI), average and minimum SpO2 levels, proportions of various sleep stages (NREM1, NREM2, NREM3, and REM), and the Oxygen Desaturation Index. PSG data were scored according to the American Academy of Sleep Medicine guidelines (17). All participants were grouped into PS and OSA groups according to OAHI. Moreover, OAHI was defined as the total number of obstructive apneas, mixed apneas, and obstructive hypopneas per hour of total sleep time. OSA diagnosis was based on OAHI ≥1 event per hour during PSG. PS was diagnosed in children with habitual snoring and OAHI <1 event per hour (18).

### 2.4 Measurement of urine indicators

On the morning after polysomnography, urine samples were collected from participants after a fast (at least 6 h)and stored at −80°C until batch assays were undertaken. The 8-OHdG concentration in urine was measured by the commercial ELISA assay kit (HCN1 + TRPV1 ELISA Kit; Jiangsu Meimian Industrial Co., Ltd., Yangchen, China.). Each reaction was performed in triplicate.

### 2.5 Cognitive assessment

To evaluate cognitive function, the PedsQL parent proxy-report was employed, encompassing four generic core scales: (1) Physical Functioning, (2) Emotional Functioning, (3) Social Functioning, and (4) School Functioning (7). A mean score of the PedsQL total score was utilized to categorize participants into two groups: the non-cognitively impaired (scoring above average) and the cognitively impaired (scoring below average).

### 2.6 Statistical analysis

All statistical analyses included in this study were conducted using SPSS 25 and R 4.3.2 software. The distribution of the continuous data was tested using the Kolmogorov–Smirnov test. The continuous variables with a normal distribution were presented as means ± standard deviation and analyzed using Student’s t-test. The continuous variables with a skewed distribution were expressed as medians (quantiles) and analyzed using non-parametric tests. Categorical variables were expressed as n (%) and analyzed using the chi-square test. The associations between urinary biomarker concentrations (8-OHdG) and parameters of PSG and cognitive dysfunctions (physical, emotional, social, and school functioning) were explored using either Pearson’s correlation or Spearman’s correlation. The diagnostic value of 8-OHdG was assessed using receiver operating characteristic (ROC). A logistic regression model was used to assess the factors influencing the cognitive status. *p*-values <0.05 were considered statistically significant.

## 3 Results

### 3.1 Participant characteristics

After inclusion and exclusion criteria, the dataset encompassed 131 participants, distributed as follows: 99 in the obstructive sleep apnea (OSA) group and 35 in the primary snoring (PS) group. Using the average score of PedsQL, the patients with OSA were divided into two groups: a non-cognitive impairment group including 43 patients, and a cognitive impairment group comprising 53 patients. The demographic, clinical characteristics, and PSG results of the groups are summarized in Tables 1, 2. No significant differences were observed in sex, age, and BMI among the three groups. In terms of PSG results, there were significant differences in Minimum SaO2, Average SaO2, OAHI, and ODI among the PS group, mild OSA group, moderate-to-severe OSA group, as well as among the PS group, OSA with cognitive impairment group, and OSA without cognitive impairment group. In addition, the proportion of NREM3 sleep stages was significantly different among the PS, non-cognitive impairment, and cognitive impairment groups.

**Table 1:** Comparisons of demographic, PSG characteristics and the urinary 8-OHdG concentration among the primary snoring, the mild and the moderate-to-severe OSA groups.

Characteristics	Primary Snoring (*n* = 35)	Mild OSA (*n* = 63)	Moderate-to-Severe OSA (*n* = 33)	*p*
Males (%)	57.14	53.97	75.76	0.106
Age (years)	7.92 ± 1.99	8.07 ± 2.36	8.16 ± 2.36	0.913
BMI (kg/m2)	16 (15.38, 20.2)	17.3 (15.4, 19.8)	17.8 (15.46, 24.7)	0.141
NREM1 (%)	5.04 ± 3.07	3.3 (1.3, 7.3)	6.08 ± 5.04	0.478
NREM2 (%)	52.39 ± 5.97	50.23 ± 8.70	48.71 ± 6.42	0.054
NREM3 (%)	23.4(19.7, 25.2)	26.25 ± 6.84	26.51 ± 6.59	0.052
REM (%)	18.72 ± 4.91	18.79 ± 4.69	18.68 ± 5.30	0.993
Minimum SaO2 (%)	91 (89, 94)	91 (88,92)	86 (78, 89)	<0.001
Average SaO2 (%)	97 (97, 97)	97 (97, 97)	96 (95, 96.5)	<0.001
OAHI	0.4 (0.3, 0.7)	2.3 (1.5, 3.5)	11.7 (6.35, 21.15)	<0.001
ODI	0.5 (0.1, 1.1)	1 (0.3, 2)	5.8 (2.4, 15.5)	<0.001
8-OHdG (pg/mL)	230.73 ± 13.82	240.94 ± 11.51	264.99 ± 7.95	<0.001

PSG, polysomnography; OSA, obstructive sleep apnea; BMI, body mass index; SaO2, arterial oxygen saturation; OAHI, obstructive apnea-hypopnea index; ODI, oxygen desaturation index.

**Table 2:** Comparisons of demographic, PSG characteristics and the urinary 8-OHdG concentration among the primary snoring, the OSA without cognitive impairment and the OSA with cognitive impairment groups.

Characteristics	Primary Snoring (*n* = 35)	OSA without CI (*n* = 43)	OSA with CI (*n* = 53)	*p*
Males (%)	57.1	62.8	60.4	0.879
Age (years)	7.93 ± 1.99	8.41 ± 2.45	7.5 (6, 9)	0.413
BMI (kg/m2)	16 (15.38, 20.2)	17.5 (15.5, 20)	17.3 (15.33, 22.6)	0.419
NREM1 (%)	5.03 ± 3.07	3.8 (1.6, 7.3)	4 (1.3, 9.65)	0.702
NREM2 (%)	52.39 ± 5.97	50.55 ± 7.38	49.02 ± 8.45	0.123
NREM3 (%)	23.4 (19.7, 25.2)	25.79 ± 6.36	26.78 ± 7.02	0.046
REM (%)	18.72 ± 4.91	18.83 ± 5.11	18.69 ± 4.73	0.99
Minimum SaO2 (%)	91 (89, 94)	90 (88, 92)	88 (84.5, 92)	0.001
Average SaO2 (%)	97 (97, 97)	97 (96, 97)	97 (96, 97)	0.022
OAHI	0.4 (0.3, 0.7)	2.4 (1.7, 5.3)	4.2 (2.3, 10.45)	<0.001
ODI	0.5 (0.1, 1.1)	1.8 (0.5, 3.4)	1.7 (0.8, 7.25)	<0.001
8-OHdG (pg/mL)	230.72 ± 13.82	244.55 (227.15, 258.55)	253.23 ± 13.69	<0.001

PSG, polysomnography; OSA, obstructive sleep apnea; CI, cognitive impairment; BMI, body mass index; SaO2, arterial oxygen saturation; OAHI, obstructive apnea-hypopnea index; ODI, oxygen desaturation index.

### 3.2 Urinary indicator

Notable significant differences were observed in urinary 8-OHdG levels among the PS, mild OSA, and moderate-to-severe OSA groups, as well as among the PS, OSA with cognitive impairment, and OSA without cognitive impairment groups (Tables 1, 2).

### 3.3 Cognitive assessment

The differences in four generic core scale scores of PedsQL between the PS and OSA groups are shown in Table 3. Compared with the patients in the PS group, the patients in the OSA group had significantly lower scores in school functioning and social functioning (all *p* < 0.05). The differences in four generic core scale scores of PedsQL between the OSA with cognitive impairment and the OSA without cognitive impairment groups are shown in Table 4. A comparison between children with and without cognitive impairment showed significant differences in school, emotional, social, and physical functioning (all *p* < 0.05).

**Table 3:** Comparisons of four generic core scales scores of PedsQL between the primary snoring and the OSA groups.

PedsQL	Primary Snoring (*n* = 35)	OSA (*n* = 96)	*p*
School functioning	75 (70, 90)	70 (55, 80)	0.014
Emotional functioning	72.69 ± 13.25	70 (55,80)	0.173
Social functioning	95 (80, 100)	85 (75, 100)	0.032
Physical functioning	87.50 (78.50, 93.75)	85.93 (68.75, 93.75)	0.089

OSA, obstructive sleep apnea.

**Table 4:** Comparisons of four generic core scales scores of PedsQL between the OSA without cognitive impairment and the OSA with cognitive impairment groups.

PedsQL	OSA without CI (*n* = 43)	OSA with CI (*n* = 53)	*p*
School functioning	80 (75, 90)	60 (47.5, 70)	<0.001
Emotional functioning	77.91 ± 13.72	60.28 ± 15.61	<0.001
Social functioning	85 (80, 100)	75.09 ± 14.66	<0.001
Physical functioning	87.50 (78.75, 93.75)	71.05 ± 13.36	<0.001

OSA, obstructive sleep apnea; CI, cognitive impairment.

### 3.4 Correlation analysis

**Figure 1:**
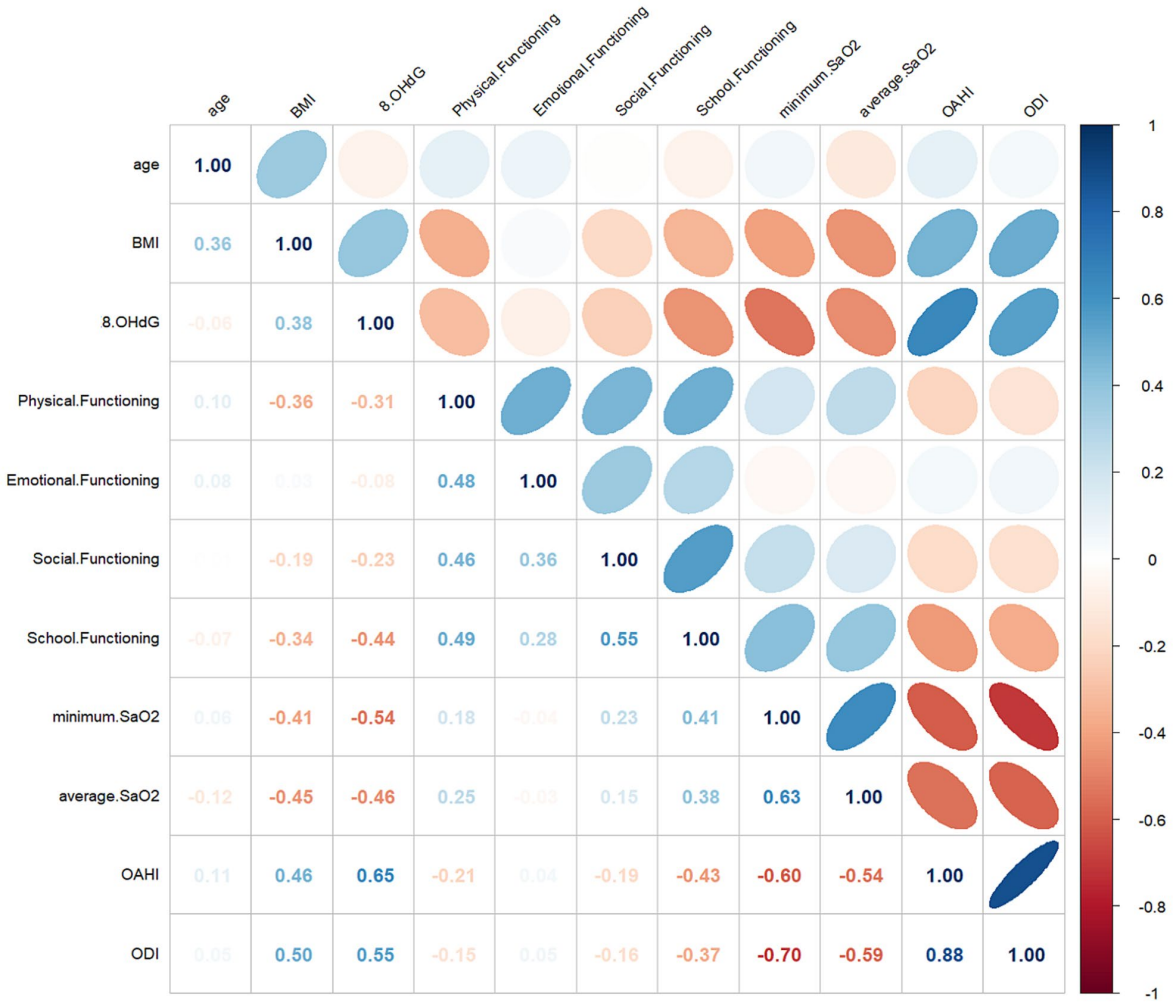
Multivariate correlations with 8-OHdG and clinical parameters. The numerical value of each cell is the spearman correlation coefficient. The baseline (r) value (white) is 0, the maximum value (blue) is 1, the light red is −0.5, and the dark red is below −0.5.

The baseline characteristics data showed a correlation between 8-OHdG and BMI (*r* = 0.38, *p* < 0.05). PSG results showed a good correlation between 8-OHdG and OAHI (*r* = 0.65, *p* < 0.001). In addition, 8-OHdG correlated with the proportion of NREM3 sleep stages (*r* = 0.173, *p* < 0.05), minimum SaO2 (*r* = −0.54, *p* < 0.001), average SaO2 (*r* = −0.46, *p* < 0.001), and ODI (*r* = 0.55, *p* < 0.001). In PedsQL, a negative correlation was observed between 8-OHdG and school functioning (*r* = −0.44, *p* < 0.001), and social functioning (*r* = −0.23, *p* < 0.05), and physical functioning (*r* = −0.31, *p* < 0.001). The specific correlation analysis results are shown in Table 5 and Figure 1.

**Table 5:** Correlation analysis between baseline characteristics, urinary indicator, behavioral and cognitive parameters.

Variables	8-OHdG	
	Correlation coefficient	*p*
Age	−0.06	0.348
BMI	0.38	0.002
NREM1 (%)	0.003	0.973
NREM2 (%)	−0.119	0.176
NREM3 (%)	0.173	0.049
REM (%)	−0.072	0.416
Minimum SaO2	−0.54	<0.001
Average SaO2	−0.46	<0.001
OAHI	0.65	<0.001
ODI	0.55	<0.001
School functioning	−0.44	<0.001
Emotional functioning	−0.08	0.357
Social functioning	−0.23	0.023
Physical functioning	−0.31	<0.001

BMI, body mass index; SaO2, arterial oxygen saturation; OAHI, obstructive apnea-hypopnea index; ODI, oxygen desaturation index.

### 3.5 Diagnostic performance of 8-OHdG

**Figure 2:**
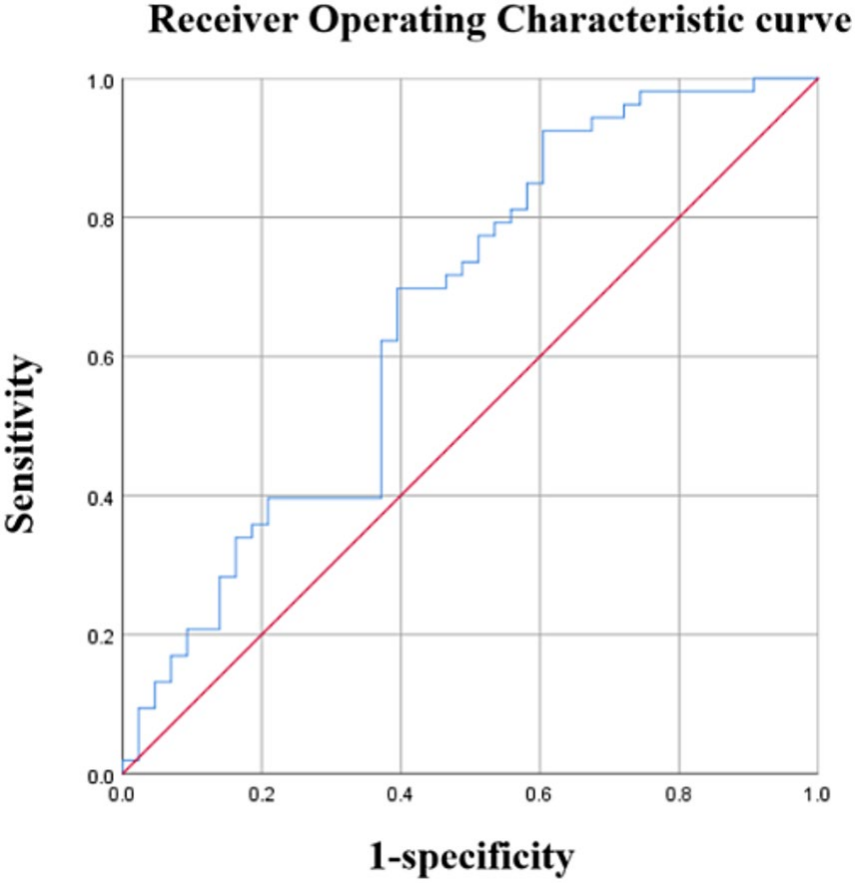
Diagnostic performance of 8-OHdG. Area under the curve (AUC) =0.661 (95% confidence interval: 0.550–0.773), *p* < 0.01.

**Figure 3:**
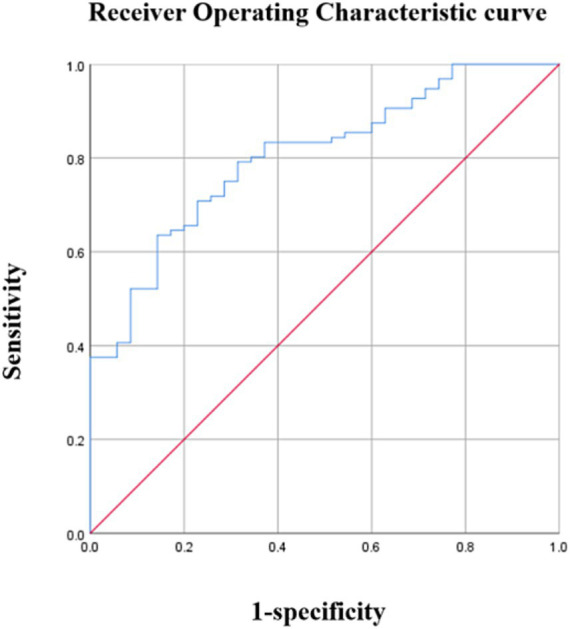
Diagnostic performance of 8-OHdG. Area under the curve (AUC) =0.804 (95% confidence interval: 0.726–0.882), *p* < 0.01.

The average PedsQL score was used to separate patients with OSA into cognitive and non-cognitive impairment groups. Urinary 8-OHdG levels were able to distinguish children with cognitive impairments from those without with an AUC of 0.661 (95%CI: 0.550–0.773) (*p* < 0.001) and a maximal Youden index of 0.303 (Figure 2). Using a cutoff value of 8-OHdG of 246.20 pg./mL resulted in 69.8% sensitivity and 60.5% specificity (Table 6). Moreover, urinary 8-OHdG levels were able to distinguish children with OSA from those experiencing PS with an AUC of 0.804 (95%CI: 0.726–0.882) (*p* < 0.001) and a maximal Youden index of 0.492 (Figure 3). Using a cutoff value of 8-OHdG of 244.225 pg/mL resulted in 63.5% sensitivity and 85.7% specificity (Table 7).

**Table 6:** Diagnostic performance of 8-OHdG.

Cutoff (pg/ml)	Sensitivity	Specificity	Youden index
246.20	0.698	0.605	0.303

**Table 7:** Diagnostic performance of 8-OHdG.

Cutoff (pg/ml)	Sensitivity	Specificity	Youden index
244.225	0.635	0.857	0.492

### 3.6 Analysis of factors influencing the cognitive status of participants

The results of correlation analysis revealed that among the PedsQL scale domains, the School Functioning score demonstrated the strongest correlation with 8-OHdG concentration. Using the median School Functioning score as the cutoff, children with OSA were stratified into school function impairment and non-impairment groups. Binary logistic regression analysis was performed with the presence of school function impairment as the dependent variable, while gender, age, BMI, 8-OHdG concentration, OAHI, DOI, and average SaO2 were included as independent variables. The analysis identified 8-OHdG concentration as an independent risk factor for school function impairment (*p* = 0.004, OR = 1.076, 95% CI: 1.024–1.131), whereas none of the other variables reached statistical significance (Table 8).

**Table 8:** Results of the binary logistic regression analysis: identify independent factors influencing school functioning in OSA.

Variables	Coefficient of determination	Standard Deviation	Wald	*p*	odds ratio	95%CI
Males	0.246	0.541	0.208	0.649	1.279	(0.443–3.694)
Age	−0.126	0.122	1.067	0.302	0.882	(0.695–1.120)
BMI	0.104	0.077	1.838	0.175	1.109	(0.955–1.289)
8-OHdG	0.073	0.026	8.276	0.004	1.076	(1.024–1.131)
OAHI	−0.03	0.061	0.238	0.626	0.971	(0.861–1.094)
ODI	−0.003	0.076	0.001	0.973	0.997	(0.859–1.158)
Average SaO2	−0.406	0.296	1.884	0.170	0.666	(0.373–1.190)

CI, confidence interval.

## 4 Discussion

The key findings of this study are as follows: (1) Urinary 8-OHdG levels were significantly higher in children with OSA than those in children with non-OSA; (2) Urinary 8-OHdG levels were negatively correlated with PedsQL scores; (3) Elevated urinary 8-OHdG levels demonstrated significant diagnostic accuracy in differentiating children with OSA from those without OSA and showed good diagnostic accuracy in distinguishing children with OSA and cognitive impairment from those without; (4) Elevated urinary 8-OHdG levels were independently associated with cognitive function impairment in children with OSA.

Our data aligns with previous research, supporting the role of oxidative stress in cognitive disorders. Children with OSA and cognitive impairment had higher 8-OHdG levels compared to those without (14). Research indicates that children with OSA exhibit higher levels of NOX activity and urinary 8-OHdG compared to controls, but these markers are lower in those without cognitive impairment. Children with OSA carrying the rs4673 polymorphism demonstrate even lower NOX activity and urinary 8-OHdG levels, suggesting a correlation between oxidative stress and cognitive function (14). Previous studies examining the association between 8-OHdG levels and the severity of OSA have predominantly focused on adults. For instance, Peres BU et al. reported that after adjusting for potential confounders such as age, sex, BMI, and statin use, the severity of OSA remained independently associated with 8-OHdG levels (15). As expected, our study findings indicate that the correlation between 8-OHdG levels and the severity of OSA holds for the pediatric population as well.

Studies have demonstrated that in OSA, the cyclical pattern of hypoxia/reoxygenation disrupts the equilibrium between oxidant and antioxidant defense systems. This imbalance triggers OS, which subsequently initiates and amplifies both peroxidation damage and inflammatory responses. Repeated episodes of airway obstruction and collapse during sleep in OSA patients lead to nocturnal chronic intermittent hypoxia (CIH), resulting in mitochondrial and endoplasmic reticulum dysfunction, excessive activation of NADPH oxidase, and reduced antioxidant capacity. This further triggers the overproduction of ROS, initiating protein, lipid, and DNA peroxidation damage and an inflammatory response. ROS can originate from various subcellular compartments, such as mitochondria, cell membranes, endoplasmic reticulum, peroxisomes, and lysosomes (19–21). Among these, mitochondria and cell membranes are the principal sources of ROS in brain cells (19). Mitochondria play a crucial role in ROS production within hippocampal neurons (22). During normal metabolic processes, oxygen can generate ROS; however, in the context of CIH, mitochondrial oxidative metabolism is disrupted at complex I/III levels (19, 23), resulting in a significant increase in ROS levels. This imbalance between elevated ROS and diminished antioxidant defenses induces oxidative stress. Nanduri et al. (24) demonstrated that CIH can activate xanthine oxidase (XO) in PC12 cells, leading to XO-dependent ROS production, which subsequently raises intracellular calcium levels. This elevation activates NOX2 and calpains, thereby mediating the imbalance of hypoxia-inducible factor-1α (HIF-1α) and HIF-2α. Furthermore, elevated ROS levels in patients with obstructive sleep apnea syndrome (OSAS) may be linked to the activation of protein kinase C (PKC)-dependent NADPH oxidases (23). Additionally, other studies have corroborated that ROS in CIH elevate intracellular calcium levels, leading to PKC-dependent regulation of mammalian target of rapamycin (mTOR) activity and increased HIF-1α expression (25). This, in turn, promotes the expression of the NOX2 gene, which is responsible for NADPH oxidase (26).

8-OHdG is an oxidative derivative of deoxyguanosine, one of the primary products of DNA oxidation; it is generated when guanine bases in DNA molecules are attacked by singlet oxygen and hydroxyl radicals at the 8th carbon atom, and it is subsequently removed from the DNA chain through processes such as base excision repair, with 8-oxo-guanine DNA glycosylase functioning as a protective mechanism. Ultimately, it is excreted from the body in a free form via urine (27). Furthermore, individuals with OSA often exhibit higher serum levels of 8-OHdG compared to healthy individuals, suggesting a link between DNA oxidative damage and the occurrence and progression of OSA (15). Our study reveals a significant correlation between 8-OHdG levels and PedsQL scores, particularly with School Functioning, which aligning with previous human research. The preliminary study indicated that the most significantly altered region of brain morphology in OSA patients was the hippocampus, a component of the limbic system responsible for regulating learning and memory functions, particularly the storage of short-term memory (28). Furthermore, cerebral gray matter is closely linked to executive functions. Through diffusion tensor magnetic resonance imaging, prior research has demonstrated extensive white matter impairment in OSA patients, particularly affecting axon-related brain tissues such as the limbic system, pons, and the frontotemporal and parietal cerebral cortex (29).

In mammals, the central nervous system is incapable of storing oxygen, and neuronal function is swiftly compromised if oxygen supply is disrupted, even briefly (30). This system consists of two primary cell types: neurons and glial cells, which include astrocytes, oligodendrocytes, and microglia. Oligodendrocytes are myelin-producing cells in the central nervous system, essential for maintaining axonal integrity and ensuring the rapid and efficient transmission of bioelectric signals (31). These cells differentiate from oligodendrocyte precursor cells (OPCs). Both oligodendrocytes and OPCs are highly vulnerable to various mechanisms such as excitotoxic injury, oxidative stress, and inflammatory events (31). OPCs, in particular, are susceptible to oxidative stress (32), which can impair their growth, differentiation, and myelination by damaging nuclear DNA (nDNA) and mitochondrial DNA (mtDNA), inhibiting mitochondrial function, and interfering with epigenetic processes (32–34). This susceptibility may stem from inadequate protective mechanisms against oxidative stress in these cells (34). Additionally, Kumar et al. (35) observed damage to axons and myelin sheaths in recently diagnosed and untreated patients with obstructive sleep apnea syndrome (OSAS), affecting fibers in the basal ganglia, hippocampus, and amygdala, which are involved in cognition, memory, and autonomy. This damage may be attributed to myelin’s heightened sensitivity to hypoxia. Consequently, the functional integrity of oligodendrocytes and OPCs is crucial for the central nervous system. In summary, ROS attacks deoxyguanosine in DNA molecules, preferentially adding a hydroxyl group at the 8th carbon atom of the guanine base, forming 8-OHdG. This oxidative damage is commonly found in mitochondrial DNA and nuclear DNA. Due to the high energy demand of neural cells, they are particularly susceptible to mitochondrial dysfunction, leading to the degeneration or apoptosis of neural cells. Therefore, 8-OHdG, as a direct product of DNA damage under oxidative stress, holds value in predicting the severity of OSA patients’ conditions and the extent of cognitive impairment.

PSG is the gold standard for diagnosing OSA (18, 36). However, assessing cognitive function in children presents a complex and challenging task. Various questionnaires are employed to evaluate cognitive performance in children, such as the Wechsler Preschool and Primary Scale of Intelligence, Third Edition (WPPSI-III) (37), the Stanford-Binet, Fourth Edition (38), the Differential Ability Scales (39) and the NEPSY (40). However, there is no gold standard among them. Furthermore, subjective errors exist, they are insensitive to early cognitive impairments, and they require time and effort. Subjective biases and insufficient sensitivity to early cognitive impairments highlight the need for a more objective and time-efficient approach to neurocognitive assessment. This would facilitate risk stratification and enhance the accuracy of cognitive outcome predictions in pediatric populations. However, we observed a significant correlation between urinary 8-hydroxydeoxyguanosine (8-OHdG) and four cognitive scores assessing cognitive function. Our findings, in conjunction with empirical findings, indicate that urinary 8-OHdG may serve as a reliable biomarker for assessing oxidative DNA damage in the brain.

This study has several limitations. First, we only measured 8-hydroxy-2′-deoxyguanosine (8-OHdG) levels in a single morning urine sample collected the day after polysomnography (PSG) monitoring in children with obstructive sleep apnea (OSA). Future longitudinal studies should investigate urinary 8-OHdG levels at multiple time points and evaluate their predictive value across different sampling periods. Second, the exclusive use of the Pediatric Quality of Life Inventory (PedsQL) scale for cognitive function assessment might have led to underestimation of other coexisting cognitive impairments in OSA children. Third, the lack of urinary 8-OHdG data and cognitive function assessments from healthy controls limits comparative analysis. Finally, the small sample size and single-center design may have compromised statistical power. To address these limitations, future research should prioritize multicenter prospective studies with larger cohorts and randomized controlled trial designs. For cognitive evaluation, comprehensive neuropsychological testing should supplement the current subjective assessment tools to better characterize cognitive dysfunction in pediatric OSA patients. Additionally, objective neuroimaging techniques such as cranial magnetic resonance imaging (MRI) should be incorporated alongside existing subjective measures. The integration of multiple assessment modalities will enhance diagnostic accuracy for disease severity stratification and is crucial for elucidating the relationship between urinary 8-OHdG levels and cognitive dysfunction in children with OSA.

## 5 Conclusion

Urinary 8-OHdG levels in children with OSA were elevated compared to those with PS. Urinary 8-OHdG levels were associated with school, social, and physical functioning, potentially reflecting the severity of cognitive impairment in children with OSA.

## Data Availability

The raw data supporting the conclusions of this article will be made available by the authors, without undue reservation.
